# Analysis of independent risk factors for progression of different degrees of diabetic retinopathy as well as non-diabetic retinopathy among type 2 diabetic patients

**DOI:** 10.3389/fnins.2023.1143476

**Published:** 2023-04-06

**Authors:** Zheng Li, Jie Tong, Chang Liu, Mingqiong Zhu, Jia Tan, Guoping Kuang

**Affiliations:** ^1^Department of Ophthalmology, The First People’s Hospital of Chenzhou, Chenzhou, Hunan, China; ^2^Department of Ophthalmology, The Affiliated Chenzhou Hospital, Hengyang Medical School, University of South China, Chenzhou, Hunan, China; ^3^Department of Spinal Surgery, The First People’s Hospital of Chenzhou, Chenzhou, Hunan, China; ^4^Department of Spinal Surgery, The Affiliated Chenzhou Hospital, Hengyang Medical School, University of South China, Chenzhou, Hunan, China; ^5^Department of Endocrinology, The First People’s Hospital of Chenzhou, Chenzhou, Hunan, China; ^6^Department of Ophthalmology, Xiangya Hospital of Central South University, Changsha, Hunan, China

**Keywords:** diabetes mellitus, retinopathy, progression, risk factor, cross-sectional study

## Abstract

**Purpose:**

To study the independent risk factors for development of different degrees of diabetic retinopathy (DR) as well as non-DR (NDR) among type 2 diabetic patients.

**Methods:**

This cross-sectional study included 218 patients with type 2 diabetes between January 2022 and June 2022. All the patients were divided into two groups: the DR group and the NDR group. The DR group was subdivided into the mild, moderate and severe non-proliferative DR (NPDR) group and the proliferative DR (PDR) group. Data recorded for all patients included age, gender, duration of diabetes, blood pressure, glycated hemoglobin (HbA1c), fasting blood glucose (FBG), blood lipids, best corrected visual acuity (BCVA), intraocular pressure (IOP), axial length (AL), anterior chamber depth (ACD), and renal function. Logistic regression methods were used to analyze the risk factors for DR.

**Results:**

The prevalence of DR in type 2 diabetes was 28.44%. The duration of diabetes, age, mean arterial pressure (MAP), HbA1c, FBG, urinary albumin/creatinine ratio (UACR), BCVA, AL, and ACD were significantly different between the DR and the NDR groups (*p* < 0.05). Multivariate logistic regression analysis identified age, FBG, UACR, and AL as the independent risk factors for DR (OR = 0.843, 2.376, 1.049, 0.005; *p* = 0.034, 0.014, 0.016, *p* < 0.001).

**Conclusion:**

Young age, short AL, higher levels of FBG and UACR were the independent risk factors for the progression of DR in type 2 diabetes.

## Introduction

The International Diabetes Federation (IDF) estimated the global burden of diabetes mellitus (DM) to be 463 million in 2019 and projected it to be 700 million by 2045. Diabetic retinopathy (DR) is a common and severe microvascular complication of DM, and one of the major diseases causing blindness in adults. In recent years, the prevalence and blindness rate of DR have significantly increased, seriously threatening the quality of life of patients with diabetes. With a rapidly aging global population, increasing lifespan of people living with DM, and lifestyle changes leading to an increased risk for DM, a higher burden of DR and demand for eye care and treatment are expected (Ting et al., 2016; Teo et al., 2021).

Several studies have shown that the risks of DR onset and its progression are modified by a variety of systemic and ocular factors. Age, male sex, hypertension, duration of diabetes, diabetic neuropathy, diabetic nephropathy, fasting blood glucose, serum total cholesterol, serum triglyceride, and glycated hemoglobin (HbA1c) are risk factors for diabetic retinopathy (Ting et al., 2016; Yin et al., 2020). Longer duration of diabetes has a higher likelihood of predicting DR. However, few studies have addressed the issue of whether there are differences in the risk factors for the duration of diabetes and age between the groups with different degrees of DR. We suppose that the risk factors of DR may be inconsistent under different courses of T2DM. Currently, the treatment of DR is mainly to prevent or delay disease progression. Early detection and intervention of risk factors can reduce the progression of DR. Hence, it is of great significance to focus on the risk factors of DR at an early stage of T2DM.

In addition, more attention should be paid to the relationship between Myopia and DR. High myopia has been suggested to have a protective effect against DR (Lin et al., 2020). However, it remains unclear whether the protective association of myopia with DR is related to the long axial length or to other components, such as ACD. Hence, it is critical to explore the independent risk factors for progression of different degrees of DR as well as NDR and target the high-risk factors for active and effective prevention.

## Materials and methods

### Materials

This cross-sectional study analyzed 218 consecutive patients with type 2 diabetes admitted to the Endocrinology Department of the First People’s Hospital of Chenzhou between January 2022 and June 2022. The study was conducted in accordance with the tenets of the Declaration of Helsinki and was approved by the hospital ethics committee (No. 2022025). Written informed consent was provided by all patients before joining the study.

Inclusion criteria were as follows: (1) age ≥ 18 years. (2) Patients with type 2 diabetes who met the diagnostic criteria (Chinese Diabetes Society, 2020) of the Chinese guidelines for the prevention and treatment of type 2 diabetes (2020 Edition) as follows: typical diabetes symptoms plus plasma glucose level ≥ 11.1 mmol·L^−1^ at any time, or fasting plasma glucose level ≥ 7.0 mmol · L^−1^, or blood glucose level ≥ 11.1 mmol · L^−1^ 2 h after glucose load. (3) Patients or family members gave informed consent.

Exclusion criteria were as follows: (1) patients with type 1 diabetes mellitus (DM), gestational DM, ketoacidosis and hyperosmolar diabetic acidosis. (2) Patients with complications such as DM with iatrogenic Cushing’s syndrome, Sheehan syndrome, acute purulent tonsillitis, and granulocytopenia that could affect the results of the risk factor analysis. (3) Fundus examination was affected by refractive media opacities such as retinal detachment, keratopathy, and cataract. (4) Non-diabetes mellitus-induced renal dysfunction disease. (5) A history of glaucoma, ocular trauma, or ocular surgery was present.

### Methods

Data recorded for all patients included: (1) Name, gender, age, diabetes duration, glycemic control, etc. (2) Blood pressure: systolic blood pressure (SBP), diastolic blood pressure (DBP) at rest. Mean arterial pressure (MAP) was calculated as: MAP = (SBP + 2 × DBP)/3. (3) Early morning fasting blood samples were collected to measure serum triglycerides (TG), low-density lipoprotein cholesterol (LDL-C), glycated hemoglobin (HbA1c), and fasting blood glucose (FBG). (4) Twenty four hour urine samples were collected from patients to determine urinary albumin/creatinine ratio (UACR). (5) Blood sample and ocular examinations were performed on the same day in this study. Ophthalmic examination included best corrected visual acuity (BCVA), non-contact tonometry (model CT-80A; TOPCON, Japan), slit-lamp examination, ocular biological measurements (ocular axis, anterior chamber depth). Anterior chamber depth (ACD) and axial length (AL) of the globe using an IOLMaster (version 5.02; Carl Zeiss Meditec, Germany), Fundus examination after dilated pupils as well as fundus photography (model CR-2AF; Canon, Japan), fluorescein fundus angiography (FFA) (model HRA-II; Heidelberg, Germany) was performed on patients with confirmed DR as permitted by voluntary and systemic conditions.

Criteria for DR diagnosis and staging were according to the 2017 SED/SERV consensus guidelines (Corcóstegui et al., 2017). Those with no DR in both eyes were classified as the non-DR (NDR) group, those with DR diagnosed in one or both eyes were classified as the DR group, and those with discordant lesion degrees in both eyes were grouped by more severe lesion degrees. The DR group was further divided into the mild, moderate and severe non-proliferative DR (NPDR) group as well as the proliferative DR (PDR) group.

### Statistical analysis

Descriptive statistical results are presented as mean ± standard deviation (SD). Counting data were expressed as frequencies and percentages, and comparisons between groups were performed using chi-squared test. Continuous variables were reported as means and standard deviations, which were compared between groups using independent samples *t*-tests. Comparisons of means between more than two groups were performed by one-way analysis of variance (ANOVA). Univariate logistic regression analysis was used to evaluate the related risk factors for the occurrence of DR. After controlling the confounding bias, multivariate logistic regression analysis was performed to clarify the independent risk factors for the occurrence of DR. A *value of p* <0.05 was considered statistically significant. Statistical analysis was performed using SPSS 16.0.

## Results

### General characteristics of study participants

The general characteristics of the 218 patients are shown in Table 1. In this study, a total of 51 patients (51 eyes) with DR received FFA examination and a total of 30 patients (30 eyes) with DR received previous retina laser treatment. The prevalence of DR among the 218 patients was found to be 28.44%. The prevalence of NPDR was 50.3% (5.50% mild, 7.80% moderate and 8.26% severe), and the prevalence of PDR was 6.88%. Diabetes duration (DD) and age were compared between the NDR and DR groups, and the difference was statistically significant (*t* = 9.171, −4.276, *p* < 0.01). The MAP, HbA1c, FBG, BCVA, and UACR were significantly higher in the DR group than in the NDR group, while the AL and ACD were significantly lower in the DR group than in the NDR group (*p* < 0.05).

**Table 1:** Comparison of various clinical parameters between the DR group and the NDR group.

	DR group (*n* = 62)	NDR group (*n* = 156)	*t/*χ^2^	*P*
DD, year (mean ± SD)	10.10 ± 3.59	5.94 ± 2.76	9.171	<0.001*
Age, year (mean ± SD)	60.69 ± 9.70	66.65 ± 9.09	−4.276	<0.001*
Gender (male/femle, *n*)	37/25	91/65	0.033	0.856^†^
MAP (mmHg)	90.82 ± 9.58	86.20 ± 8.07	3.605	<0.001*
HbA1c (%)	7.64 ± 0.90	6.50 ± 0.79	9.150	<0.001*
FBG (mmol·L^−1^)	9.74 ± 1.32	7.78 ± 1.38	9.510	<0.001*
TG (mmol·L^−1^)	1.89 ± 0.16	1.86 ± 0.14	1.820	0.070*
LDL-C (mmol·L^−1^)	3.64 ± 0.33	3.57 ± 0.31	1.621	0.106*
UACR (mg·g^−1^)	260.29 ± 45.44	190.99 ± 14.08	17.139	<0.001*
AL (mm)	22.85 ± 0.41	24.12 ± 1.06	−9.159	<0.001*
ACD (mm)	2.79 ± 0.21	2.90 ± 0.17	−3.977	<0.001*
Mean IOP (mmHg)	14.74 ± 3.01	14.86 ± 2.76	−0.275	0.784*
Hypertension [n (%)]	30 (48.38)	42 (26.92)	9.241	0.002^†^
BCVA [n (%)]			7.887	0.019^†^
>0.1	40 (64.52)	120 (76.92)		
0.02 ~ 0.1	17 (27.42)	34 (21.79)		
<0.02	5 (8.06)	2 (1.28)		

* Independent sample *t*-test.

^†^Chi-squared test.

DD, Diabetes duration; MAP, mean arterial pressure; HbA1c, glycated hemoglobin; FBG, fasting blood glucose; TG, triglycerides; LDL-C, low-density lipoprotein cholesterol; UACR, urinary albumin/creatinine ratio; AL, axial length; ACD, anterior chamber depth; IOP, intraocular pressure; BCVA, best corrected visual acuity.

### Association between age, diabetes duration, and DR

**Figure 1:**
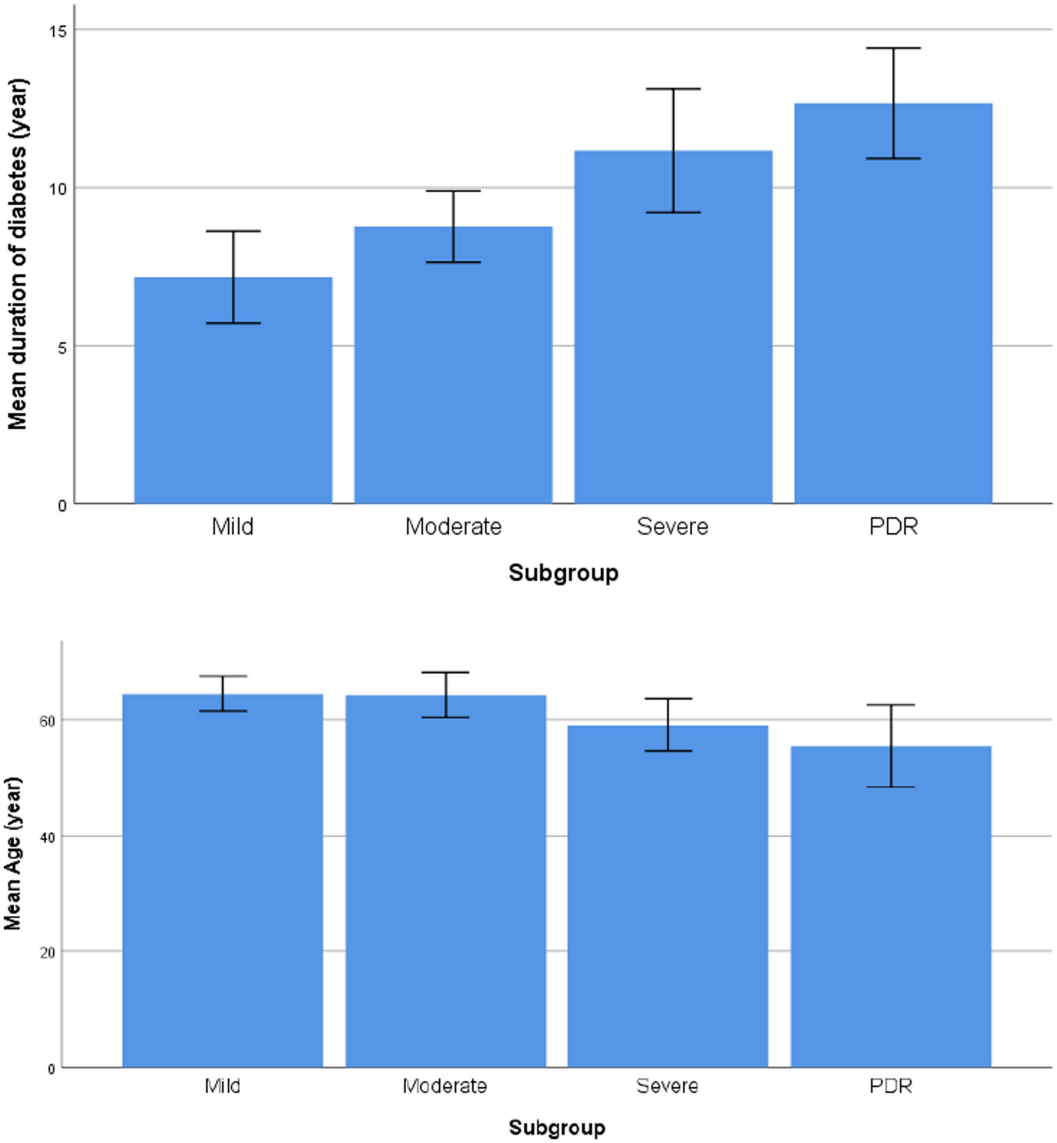
Comparison of the duration of diabetes and age between the groups with different degrees of NPDR and PDR.

Comparison of the duration of diabetes and age between the groups with different degrees of NPDR and PDR showed that with the duration of diabetes increases, the younger the age of onset, the higher the risk of progression of DR; the patients in the PDR group had a longer mean duration of diabetes and a younger mean age than the patients in the NPDR group (*F* = 9.111, 3.352, *p* < 0.001, *p* = 0.025, respectively), as shown in Table 2 and Figure 1.

**Table 2:** Comparison of the basic data of each subgroup of DR patients.

	Number of cases	DD, year (mean ± SD)	Age, year (mean ± SD)
**NPDR group**			
Mild	12	7.17 ± 2.29	64.50 ± 4.70
Moderate	17	8.76 ± 2.19	64.29 ± 7.63
Severe	18	11.17 ± 3.93	59.11 ± 9.04
**PDR group**	15	12.67 ± 3.15	55.47 ± 12.90
*F-*value		9.111	3.352
*p-*value		<0.001	0.025

NPDR, non-proliferative DR; PDR, proliferative DR.

### Risk factor analysis for DR

Univariate logistic regression analysis showed that DD, age, MAP, HbA1c, FBG, UACR, AL, and ACD were significantly associated with the development of DR (*p* < 0.05). TG and LDL-C were not associated with the occurrence of DR (*p* > 0.05), as shown in Table 3.

**Table 3:** Univariate logistic regression analysis of risk factors for DR.

	Partial regression coefficients	SD	Wald statistic	*OR*	95% *CI*	*P*
DD	0.410	0.063	42.144	1.506	1.331 ~ 1.705	<0.001
Age	−0.067	0.017	15.310	0.935	0.904 ~ 0.967	<0.001
MAP	0.059	0.017	11.607	1.060	1.025 ~ 1.097	0.001
HbA1c	1.543	0.234	43.379	4.679	2.956 ~ 7.406	<0.001
FBG	0.912	0.138	43.787	2.490	1.900 ~ 3.262	<0.001
TG	1.817	1.010	3.239	6.155	0.851 ~ 44.539	0.072
LDL-C	0.762	0.473	2.597	2.142	0.848 ~ 5.412	0.107
UACR	0.065	0.010	41.991	1.067	1.046 ~ 1.088	<0.001
AL	−7.053	1.036	46.396	0.001	0 ~ 0.007	<0.001
ACD	−2.939	0.788	13.921	0.053	0.011 ~ 0.248	<0.001

DD, Diabetes duration; MAP, mean arterial pressure; HbA1c, glycated hemoglobin; FBG, fasting blood glucose; TG, triglycerides; LDL-C, low-density lipoprotein cholesterol; UACR, urinary albumin/creatinine ratio; AL, axial length; ACD, anterior chamber depth.

The risk factors for DR such as DD, age, MAP, HbA1c, FBG, UACR, TG, LDL-C, AL, and ACD were included as independent variables in the multivariate logistic regression analysis. The results showed that age, FBG, UACR, and AL were independent risk factors for the progression of DR (*p* = 0.034, 0.014, 0.016, and *p* < 0.001, respectively), as shown in Table 4.

**Table 4:** Multivariate logistic regression analysis of risk factors for DR.

Independent variable	*OR*	95% *CI*	*P*
DD	1.293	0.778 ~ 2.149	0.321
Age	0.843	0.720 ~ 0.987	0.034
MAP	1.057	0.930 ~ 1.202	0.397
HbA1c	1.199	0.293 ~ 4.914	0.801
FBG	2.376	1.193 ~ 4.731	0.014
TG	0.002	0 ~ 12.360	0.165
LDL-C	0.300	0.005 ~ 17.730	0.563
UACR	1.049	1.009 ~ 1.091	0.016
AL	0.005	0 ~ 0.078	<0.001
ACD	0.295	0.001 ~ 103.532	0.683

DD, Diabetes duration; MAP, mean arterial pressure; HbA1c, glycated hemoglobin; FBG, fasting blood glucose; TG, triglycerides; LDL-C, low-density lipoprotein cholesterol; UACR, urinary albumin/creatinine ratio; AL, axial length; ACD, anterior chamber depth.

## Discussion

In this study, the results showed that the prevalence of DR was 28.44%, with predominance of moderate and severe NPDR. As the duration of diabetes increases, the younger the age of onset, the higher the risk of progression of DR. The patients in the PDR group had a longer mean duration of diabetes and a younger mean age than the patients in the NPDR group. The severity and prevalence of disease increased with Diabetes duration (DD), which was similar to the results of previous studies (Song et al., 2018; Yin et al., 2020). Numerous studies have shown that DR results from a combination of multiple factors, mainly related to blood glucose, blood pressure, lipids, DD, age, renal function and other factors (Song et al., 2018; Teo et al., 2021). Our results showed that DD, age, MAP, HbA1c, FBG, UACR, AL, and ACD were significantly different between the DR group and the NDR group. Patients in the NDR group had better BCVA than those in the DR group, which was related to the fundus disease in the DR Group. Moreover, age, FBG, UACR, and AL were independent risk factors for concurrent DR in patients with type 2 diabetes by multivariate logistic regression analysis. Hence, younger age, shorter AL, higher FBG and UACR levels were associated with a higher risk of concurrent DR.

Long AL may be protective against the development of DR. He et al. (2017) conducted an epidemiological investigation and found that type 2 diabetic patients with long AL had a low risk of developing DR. Man et al. (2012) stated that long AL is protective for mild, moderate, and severe DR. The mechanism of the protective effect of long AL and myopia on the development and progression of DR remains unknown, and the possible reasons are as follows: (1) Patients with longer AL have reduced retinal blood flow, slower blood velocity, and reduced vessel wall pressure, thus limiting the occurrence and progression of DR (Lim et al., 2011; Kim et al., 2018). (2) Disruption of retinal and choroidal blood vessels occurs earlier in patients with high myopia, and the drop in retinal blood perfusion prevents the formation of an abnormal hyperperfusion state that induces DR, thus reducing the risk of developing DR (Benavente-Pérez et al., 2010; Kim et al., 2018). (3) The choroid and retina become thinner with the growth of the ocular axis, and the retinal metabolic rate and oxygen demand decrease, thereby reducing the release of some inflammatory factors due to hypoxia as well as VEGF production (Sawada et al., 2011). (4) Patients with long AL and high myopia often have earlier onset of vitreous liquefaction and posterior vitreous detachment, lack of scaffolds in the vitreous that are needed for neovascular proliferation, easier diffusion of oxygen through the liquefied vitreous, reduced aggregation of promoting neovascular factors to achieve the degree of neovascularization, and therefore slowing the progression of DR (Lin et al., 2020).

Previous studies have shown that high levels of UACR, as a marker of endothelial dysfunction, may affect renal and retinal microangiopathy, and are associated with type 1 DR pathogenesis, not with type 2 DR (Pedro et al., 2010). Our results showed that UACR levels could not only assess the risk of concurrent DR in patients with type 2 diabetes but also were positively correlated with the prevalence of DR. Both glomerular and retinal vessels of the microcirculation system that share similar physiological features, with hyperglycemia driving the production of glycosylation end products, resulting in functional impairment of the vascular endothelium, enhanced oxidative stress and release of inflammatory mediators, causing disruption of the glomerular filtration membrane barrier and blood retinal barrier (Liu et al., 2022). Therefore, diabetic patients with high UACR levels should be screened for DR by fundus examination as early as possible.

Previous studies have shown that DR severity in type 2 diabetes is significantly positively correlated with TG, and LDL-C is an independent risk factor for retinopathy (Agroiya et al., 2013; Tan et al., 2018). On the contrary, some studies suggest that LDL-C has nothing to do with DR (Pang et al., 2012; Wang et al., 2012). Chang and Wu (2013) speculated that blood lipids may not directly damage endothelial tissue, only in the later stages are they pathogenic by damaging retinal vessels. Therefore, it is believed that dyslipidemia is more involved in its pathogenesis in the severe stages of DR than in the early stages. Considering the low prevalence of PDR (6.88%), our results showed that TG and LDL-C were not associated with the progression of DR.

Studies have shown that obesity, medications, and oxygen therapy are also contributing factors to DR (Thomas et al., 2012; Mcmonnies, 2015; Raum et al., 2015; Li et al., 2022). Raum et al. (2015) prospectively identified obesity (BMI ≥ 30) as a risk factor for eyesight loss in DR. However, in this cross-sectional study, the included subjects were consecutive patients hospitalized during a certain period, which was limited by geographical, dietary and lifestyle habits, with fewer patients with obesity. Therefore, a study specifically focused on the correlation between obesity and DR is needed to supplement the future study.

This study only selected UACR as an indicator to evaluate renal function, which has limitations. Studies have shown a significant association between GFR and DR in Caucasian T2DM patients, with reduced GFR increasing the risk of DR, but both associations have been inconsistently reported in Asian populations (Grunwald et al., 2012; Perol et al., 2012; Man et al., 2015; Guo et al., 2016). A cross-sectional study that included 3,301 T2DM patients showed that both proteinuria and GFR decline were significantly associated with the development of DR (Guo et al., 2016). Therefore, it may be more helpful to include indicators such as GFR and Cr in future studies to discover independent risk factors for the progression of DR.

## Conclusion

In this study, we evaluated the association between DR and systemic influencing factors, and found that young age, short axial length, and high levels of FBG and UACR were independent risk factors for the progression of DR in type 2 diabetes. Although the conclusions of this study provide a basis for clinicians to identify high-risk factors for developing DR, whether it can be used for screening DR needs to be confirmed by further large sample, multi-center studies.

## Data availability statement

The raw data supporting the conclusions of this article will be made available by the authors, without undue reservation.

## Ethics statement

The studies involving human participants were reviewed and approved by the Ethics Committee of the First People’s Hospital of Chenzhou. The patients/participants provided their written informed consent to participate in this study.

## Author contributions

ZL and JeT designed the study and drafted the manuscript. CL and MZ collected the data and carried out statistical analyses and data interpretation. GK participated in the study design and contributed to the editing of the manuscript. JaT performed the computational analysis and contributed to the preparation of the manuscript. All authors contributed to the interpretation of the data and approved the final version of the manuscript.

## Funding

This research was supported by the Science and Technology Planning Project of Chenzhou, China (No. CZKJ2016053).

## Conflict of interest

The authors declare that the research was conducted in the absence of any commercial or financial relationships that could be construed as a potential conflict of interest.

## Publisher’s note

All claims expressed in this article are solely those of the authors and do not necessarily represent those of their affiliated organizations, or those of the publisher, the editors and the reviewers. Any product that may be evaluated in this article, or claim that may be made by its manufacturer, is not guaranteed or endorsed by the publisher.

## References

[ref1] Agroiya P. Philip R. Saran S. Gutch M. Tyagi R. Gupta K. K. (2013). Association of serum lipids with diabetic retinopathy in type 2 diabetes. Indian J. Endocrinol. Metab. 17, 335–S337. doi: 10.4103/2230-8210.119637, PMID: 24251206 PMC3830352

[ref2] Benavente-Pérez A. Hosking S. L. Logan N. S. Broadway D. C. (2010). Ocular blood flow measurements in healthy human myopic eyes. Graefes Arch. Clin. Exp. Ophthalmol. 248, 1587–1594. doi: 10.1007/s00417-010-1407-9, PMID: 20502909

[ref3] Chang Y. Wu W. (2013). Dyslipidemia and diabetic retinopathy. Rev. Diabet. Stud. 10, 121–132. doi: 10.1900/RDS.2013.10.121, PMID: 24380088 PMC4063092

[ref4] Chinese Diabetes Society (2020). Guideline for the prevention and treatment of type 2 diabetes mellitus in China. Chin J Endocrinol Metab 37, 2–51. doi: 10.3760/cma.j.cn311282-20210304-00142

[ref5] Corcóstegui B. Durán S. González-Albarrán M. O. Hernández C. Ruiz-Moreno J. M. Salvador J.. (2017). Update on diagnosis and treatment of diabetic retinopathy: A consensus guideline of the working group of ocular health (Spanish society of diabetes and Spanish vitreous and retina society). J. Ophthalmol. 2017, 1–10. doi: 10.1155/2017/8234186, PMID: 28695003 PMC5488240

[ref6] Grunwald J. E. Alexander J. Ying G. S. Maguire M. Daniel E. Whittock-Martin R.. (2012). Retinopathy and chronic kidney disease in the chronic renal insufficiency cohort (CRIC) study. Arch. Ophthalmol. 130, 1136–1144. doi: 10.1001/archophthalmol.2012.1800, PMID: 22965589 PMC3719171

[ref7] Guo K. Zhang L. Zhao F. Lu J. Pan P. Yu H.. (2016). Prevalence of chronic kidney disease and associated factors in Chinese individuals with type 2 diabetes: Cross-sectional study. J. Diabetes Complicat. 30, 803–810. doi: 10.1016/j.jdiacomp.2016.03.020, PMID: 27068269

[ref8] He J. Xu X. Zhu J. Zhu B. Zhang B. Lu L.. (2017). Lens power, axial length-to-corneal radius ratio, and association with diabetic retinopathy in the adult population with type 2 diabetes. Ophthalmology 124, 326–335. doi: 10.1016/j.ophtha.2016.10.041 27993483

[ref9] Kim D. Y. Song J. H. Kim Y. J. Lee J. Y. Kim J. Yoon Y. H.. (2018). Asymmetric diabetic retinopathy progression in patients with axial anisometropia. Retina 38, 1809–1815. doi: 10.1097/IAE.0000000000002109 29547453

[ref10] Li Y. Gappy S. Liu X. Sassalos T. Zhou T. Hsu A.. (2022). Metformin suppresses pro-inflammatory cytokines in vitreous of diabetes patients and human retinal vascular endothelium. PLoS One 17:e268451:e0268451. doi: 10.1371/journal.pone.0268451, PMID: 35802672 PMC9269956

[ref11] Lim L. S. Cheung C. Y. Lin X. Mitchell P. Wong T. Y. Mei-Saw S. (2011). Influence of refractive error and axial length on retinal vessel geometric characteristics. Invest. Ophthalmol. Vis. Sci. 52, 669–678. doi: 10.1167/iovs.10-6184, PMID: 20847122

[ref12] Lin Z. Li D. Zhai G. Wang Y. Wen L. Ding X. X.. (2020). High myopia is protective against diabetic retinopathy via thinning retinal vein: A report from Fushun Diabetic Retinopathy Cohort Study (FS-DIRECT). Diab. Vasc. Dis. Res. 17:147916412094098. doi: 10.1177/1479164120940988, PMID: 32686483 PMC7510364

[ref13] Liu W. Du J. Ge X. Jiang X. Peng W. Zhao N.. (2022). The analysis of risk factors for diabetic kidney disease progression: A single-centre and cross-sectional experiment in Shanghai. BMJ Open 12:e60238. doi: 10.1136/bmjopen-2021-060238, PMID: 35768116 PMC9240884

[ref14] Man R. E. K. Sasongko M. B. Sanmugasundram S. Nicolaou T. Jing X. Wang J. J.. (2012). Longer axial length is protective of diabetic retinopathy and macular edema. Ophthalmology 119, 1754–1759. doi: 10.1016/j.ophtha.2012.03.021, PMID: 22627119

[ref15] Man R. E. K. Sasongko M. B. Wang J. J. MacIsaac R. Wong T. Y. Sabanayagam C.. (2015). The association of estimated glomerular filtration rate with diabetic retinopathy and macular edema. Invest. Ophthalmol. Vis. Sci. 56, 4810–4816. doi: 10.1167/iovs.15-16987 26218909

[ref16] Mcmonnies C. W. (2015). Hyperbaric oxygen therapy and the possibility of ocular complications or contraindications. Clin. Exp. Optom. 98, 122–125. doi: 10.1111/cxo.12203 25308346

[ref17] Pang C. Jia L. Jiang S. Liu W. Hou X. Zuo Y.. (2012). Determination of diabetic retinopathy prevalence and associated risk factors in Chinese diabetic and pre-diabetic subjects: Shanghai diabetic complications study. Diabetes Metab. Res. Rev. 28, 276–283. doi: 10.1002/dmrr.1307, PMID: 22139892

[ref18] Pedro R. Ramon S. Marc B. Juan F. Isabel M. (2010). Prevalence and relationship between diabetic retinopathy and nephropathy, and its risk factors in the North-East of Spain, a population-based study. Ophthalmic Epidemiol. 17, 251–265. doi: 10.3109/09286586.2010.498661 20642348

[ref19] Perol J. Balkau B. Guillausseau P. J. Massin P. (2012). A study of the 3-year incidence of diabetic retinopathy in a French diabetic population seen at Lariboisière Hospital, Paris. Diabetes Metab. 38, 225–229. doi: 10.1016/j.diabet.2012.01.001, PMID: 22386834

[ref20] Raum P. Lamparter J. Ponto K. A. Peto T. Hoehn R. Schulz A.. (2015). Correction: Prevalence and cardiovascular associations of diabetic retinopathy and maculopathy: Results from the gutenberg health study. PLoS One 10:e139527. doi: 10.1371/journal.pone.0139527, PMID: 26406582 PMC4583426

[ref21] Sawada O. Miyake T. Kakinoki M. Sawada T. Kawamura H. Ohji M. (2011). Negative correlation between aqueous vascular endothelial growth factor levels and axial length. Jpn. J. Ophthalmol. 55, 401–404. doi: 10.1007/s10384-011-0027-1, PMID: 21607685

[ref22] Song P. Yu J. Chan K. Y. Theodoratou E. Rudan I. (2018). Prevalence, risk factors and burden of diabetic retinopathy in China: A systematic review and meta-analysis. J. Glob. Health 8:010803. doi: 10.7189/jogh.08.010803, PMID: 29899983 PMC5997368

[ref23] Tan G. S. Gan A. Sabanayagam C. Tham Y. C. Neelam K. Mitchell P.. (2018). Ethnic differences in the prevalence and risk factors of diabetic retinopathy. Ophthalmology 125, 529–536. doi: 10.1016/j.ophtha.2017.10.026 29217148

[ref24] Teo Z. L. Tham Y. Yu M. Chee M. L. Rim T. H. Cheung N.. (2021). Global prevalence of diabetic retinopathy and projection of burden through 2045. Ophthalmology 128, 1580–1591. doi: 10.1016/j.ophtha.2021.04.027, PMID: 33940045

[ref25] Thomas R. L. Dunstan F. Luzio S. D. Roy Chowdury S. Hale S. L. North R. V.. (2012). Incidence of diabetic retinopathy in people with type 2 diabetes mellitus attending the Diabetic Retinopathy Screening Service for Wales: Retrospective analysis. BMJ 344:e874. doi: 10.1136/bmj.e874, PMID: 22362115 PMC3284424

[ref26] Ting D. S. W. Cheung G. C. M. Wong T. Y. (2016). Diabetic retinopathy: Global prevalence, major risk factors, screening practices and public health challenges: A review. Clin. Exp. Ophthalmol. 44, 260–277. doi: 10.1111/ceo.12696, PMID: 26716602

[ref27] Wang S. Xu L. Jonas J. B. You Q. S. Wang Y. X. Yang H. (2012). Dyslipidemia and eye diseases in the adult Chinese population: The Beijing eye study. PLoS One 7:e26871. doi: 10.1371/journal.pone.0026871, PMID: 22128290 PMC3419255

[ref28] Yin L. Zhang D. Ren Q. Su X. Sun Z. (2020). Prevalence and risk factors of diabetic retinopathy in diabetic patients. Medicine 99:e19236. doi: 10.1097/MD.0000000000019236, PMID: 32118727 PMC7478682

